# Supplementary material for: Occurrence of Hydroxytyrosol, Tyrosol and Their Metabolites in Italian Cheese
Source: Molecules. 2023 Aug 23;28(17):6204. doi: 10.3390/molecules28176204 (PMC10488946; doi:10.3390/molecules28176204)
Supplement: Supplementary file 1 [file molecules-28-06204-s001.zip › molecules-2539035-supplementary.pdf]

Occurrence of Hydroxytyrosol, Tyrosol and Their Metabolites in Italian Cheese

Danilo Giusepponi [1], Carolina Barola [1], Elisabetta Bucaletti [1], Simone Moretti [1], Fabiola Paoletti [1], Andrea Valiani [1], Raffaella Branciari [2] and Roberta Galarini [1,*]

[1] Istituto Zooprofilattico Sperimentale dell'Umbria e delle Marche "Togo Rosati", Via G. Salvemini 1, 06126 Perugia, Italy; d.giusepponi@izsum.it (D.G.); c.barola@izsum.it (C.B.); e.bucaletti@izsum.it (E.B.); s.moretti@izsum.it (S.M.); f.paoletti@izsum.it (F.P.); a.valiani@izsum.it (A.V.)

[2] Department of Veterinary Medicine, University of Perugia, Via San Costanzo 4, 06126 Perugia, Italy; raffaella.branciari@unipg.it

* Correspondence: r.galarini@izsum.it; Tel.: +39-075-343272; Fax: +39-075-3433060

Table S1 – Moisture contents and concentrations of phenylethanoids in commercial cheeses (continued)

Sample ID	Cheese type	Moisture (%)	HT-4-S[1] (µg kg^{-1} dw)	HT-3-S[1] (µg kg^{-1} dw)	T-S[1] (µg kg^{-1} dw)	T[1] (µg kg^{-1} dw)	HT[1] (µg kg^{-1} dw)	Sum (µg kg^{-1} dw)
**1**	Ewe cheese	23.43	128	84	12	ND	ND	224
**2**		36.55	152	113	8.9	108	ND	382
**3**		38.75	188	142	8.6	116	ND	455
**4**		38.92	278	193	16	ND	ND	487
**5**		30.81	332	117	ND	ND	ND	450
**6**		36.06	345	153	ND	ND	ND	498
**7**		36.75	413	235	ND	ND	ND	648
**8**		17.66	204	92	ND	ND	ND	296
**9**		38.09	278	215	13	65	ND	570
**10**		28.11	164	134	9.0	ND	17	340
**11**		36.87	91	75	11	54	ND	231
**12**		40.19	295	138	ND	ND	ND	433
**13**		29.94	192	222	24	57	ND	496
**14**		32.07	213	168	7.1	ND	ND	388
**15**		31.47	816	263	ND	51	ND	1130
**16**		27.73	242	184	33	228	12	710
**17**		37.98	208	165	11	273	8.8	674
**18**		37.04	176	130	15	91	ND	412
**19**		33.33	585	176	ND	ND	ND	760

Table S1 – Moisture contents and concentrations of phenylethanoids in commercial cheeses

Sample ID	Cheese type	Moisture (%)	HT-4-S[1] (μg kg^{-1} dw)	HT-3-S[1] (μg kg^{-1} dw)	T-S[1] (μg kg^{-1} dw)	T[1] (μg kg^{-1} dw)	HT[1] (μg kg^{-1} dw)	Sum (μg kg^{-1} dw)
**1**	Goat cheese	63.66	19	12	7.8	ND	ND	40
**2**		65.50	29	10	14	2296	ND	2349
**3**		50.82	52	23	12	1370	ND	1456
**4**		38.05	12	ND	9.5	74	ND	95
**1**	Cow cheese	40.55	22	9	15	362	ND	409
**2**		39.77	9.1	ND	6.2	130	ND	145
**3**		41.04	12	ND	12	292	ND	317
**4**		40.67	27	11	18	800	ND	855
**5**		45.73	ND	ND	28	1593	ND	1621
**6**		31.33	ND	ND	30	215	ND	246
**7**		36.57	5.2	ND	29	ND	ND	34
**8**		23.49	ND	ND	24	ND	ND	24
**9**		33.83	5.3	ND	9.9	ND	ND	15
**10**		30.83	ND	ND	6.5	ND	ND	6.5
**11**		28.92	ND	ND	ND	ND	ND	-
**12**		28.17	ND	ND	ND	60	ND	60
**13**		26.77	ND	ND	ND	ND	ND	-

[1]ND: Not Detected (< LOD); Results <LOD were placed equal to ½ LOD for statistical elaboration

Table S2 – Chromatographic conditions

Time (min)	A (%) aqueous phase (2 mM ammonium acetate)	B (%) Methanol	Curve
0	100	0	5
2	100	0	5
6	90	10	5
13	30	70	5
15	30	70	5
15.5	0	100	5
18	0	100	5
18.5	100	0	5
22	100	0	5

Table S3 - Formulas, structures and monitored ions of analytes

Analyte	Structure	Formula	RT (min)	Theoretical (*m/z*)	$[M-H]^-$ (*m/z*)	Fragment 1 (*m/z*)	Fragment 2 (*m/z*)
**Tyrosol Glucuronide (T-G)**	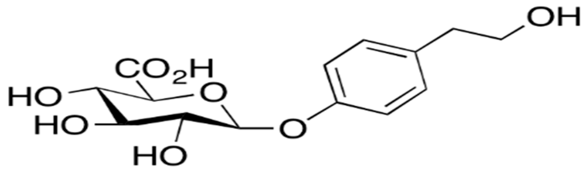	C14H18O8	6.1	314.1001	313.0929	175.0239	137.0594
**Hydroxytyrosol-3-Glucuronide (HT-3-G)**	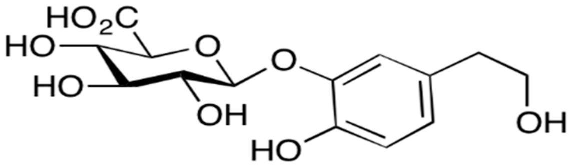	C14H18O9	6.8	330.0951	329.0878	175.0237	153.0542
**Hydroxytyrosol Sulphate (HT-S)**	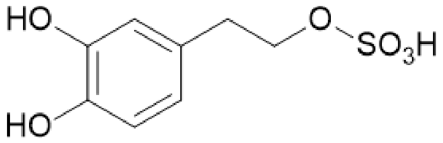	C8H10O6S	6.8	234.0198	233.0125	153.0544	123.0436
**HT-4-S**	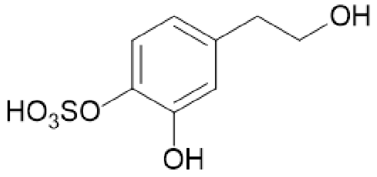	C8H10O6S	7.6	234.0198	233.0125	153.0544	123.0436

**HT-3-S**	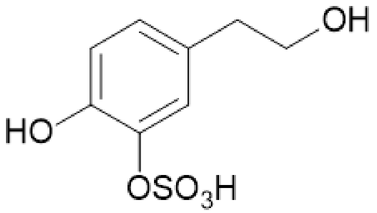	C8H10O6S	7.8	234.0198	233.0125	153.0544	123.0436
**Tyrosol sulphate (T-S)**	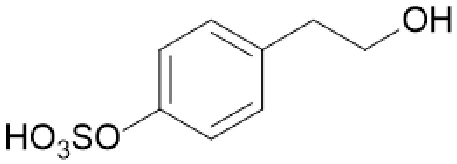	C8H10O5S	8.2	218.0249	217.0176	137.0596	119.0489
**Hydroxy Tyrosol (HT)**	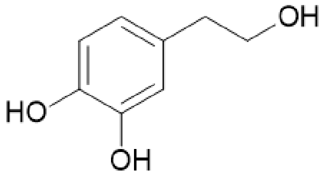	C8H10O3	8.9	154.0629	153.0557	123.0436	108.0200
**HT-d4**	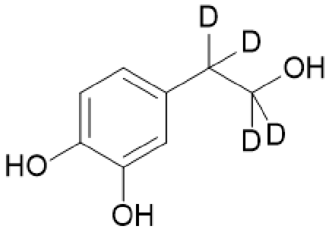	C8H6D4O3	8.9	158.0881	157.0808	-	-

**Tyrosol (T)**	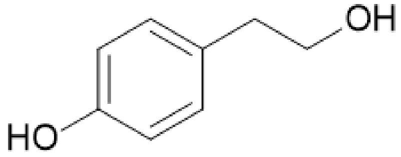	C8H10O2	10.2	138.0675	137.0608	119.0488	106.0409

Table S4 –Collected commercial cheese samples

Cheese	Sample ID	Producer	Moisture (%)	Classification[1]	Cheese	Sample ID	Producer (Italian region)	Moisture (%)	Classification of cheese[1]
Ewe cheese	1	Farm A (Umbria)	23.43	Very hard					
	2	Farm A (Umbria)	36.55	Semihard	Goat cheese	1	Farm I (Venetian)	63.66	Fresh /curd
	3	Farm A (Umbria)	38.75	Semihard		2	Farm J (Umbria)	65.50	Fresh/curd
	4	Farm A (Umbria)	38.92	Semihard		3	Farm J (Umbria)	50.82	Soft
	5	Farm A (Umbria)	30.81	Hard		4	Farm J (Umbria)	38.05	Semihard
	6	Farm A (Umbria)	36.06	Semihard					
	7	Farm A (Umbria)	36.75	Semihard	Cow cheese	1	Farm J (Umbria)	40.55	Soft
	8	Farm A (Umbria)	17.66	Very hard		2	Farm J (Umbria)	39.77	Semihard
	9	Farm B (Umbria)	38.09	Semihard		3	Farm K (Umbria)	41.04	Soft
	10	Farm C (Tuscany)	28.11	Hard		4	Farm A (Umbria)	40.67	Soft
	11	Farm C (Tuscany)	36.87	Semihard		5	Farm L (Umbria)	45.73	Soft
	12	Farm C (Tuscany)	40.19	Soft		6	Farm M (Venetian)	31.33	Hard
	13	Farm D (Tuscany)	29.94	Hard		7	Farm N (Piedmont)	36.57	Semihard
	14	Farm E (Tuscany)	32.07	Hard		8	Farm O (Venetian)	23.49	Very hard
	15	Farm E (Tuscany)	31.47	Hard		9	Farm P (Emilia Romagna)	33.83	Hard
	16	Farm F (Emilia Romagna)	27.73	Hard		10	Farm P (Emilia Romagna)	30.83	Hard
	17	Farm F (Emilia Romagna)	37.98	Semihard		11	Farm P (Emilia Romagna)	28.92	Hard
	18	Farm G (Sardinia)	37.04	Semihard		12	Farm P (Emilia Romagna)	28.17	Hard
	19	Farm H (Lazio)	33.33	Hard		13	Farm P (Emilia Romagna)	26.77	Hard

[1]Cheese classification is based on rheological properties [46]

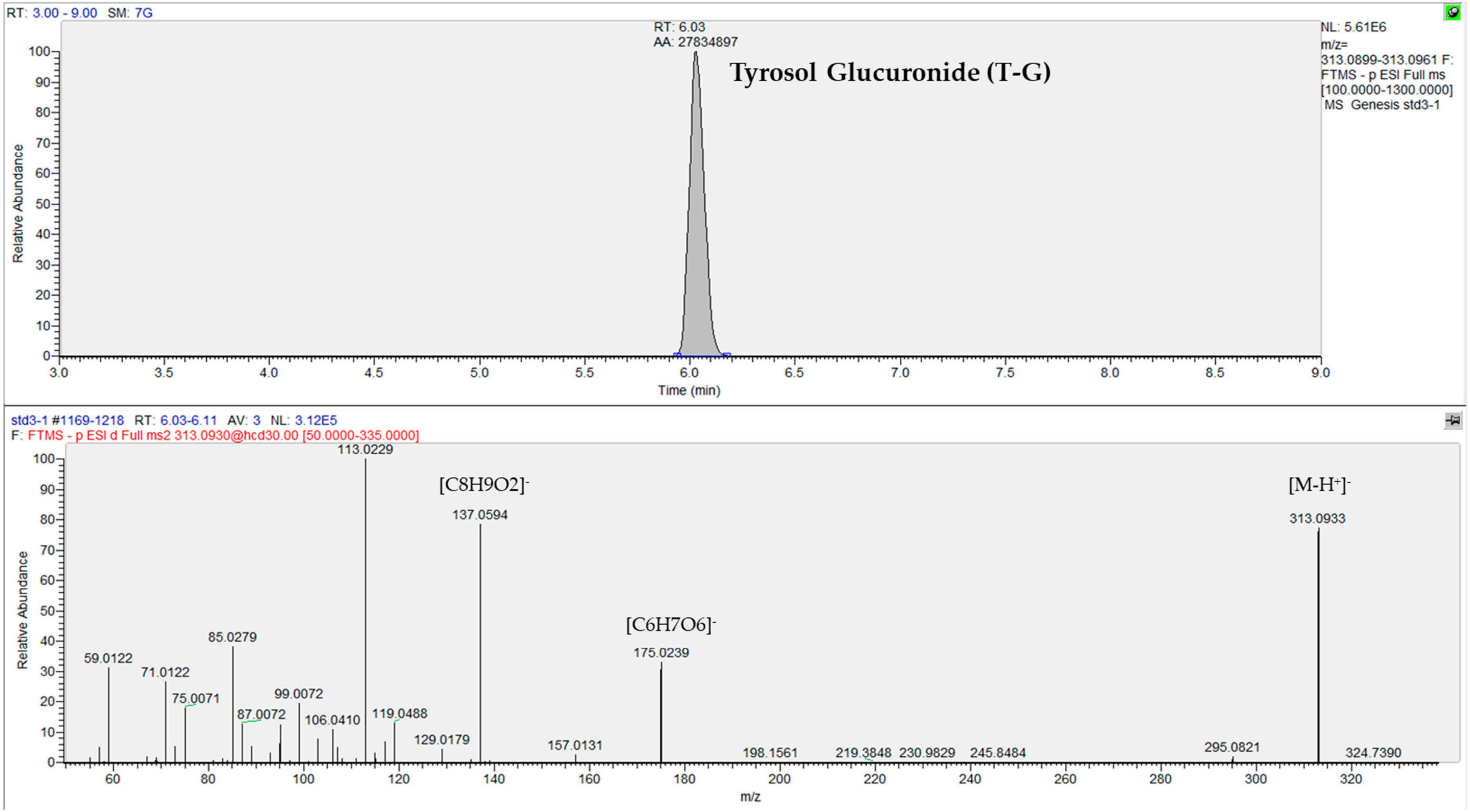

Figure S1 – LC-Q-Orbitrap chromatogram and MS² spectrum of T-G

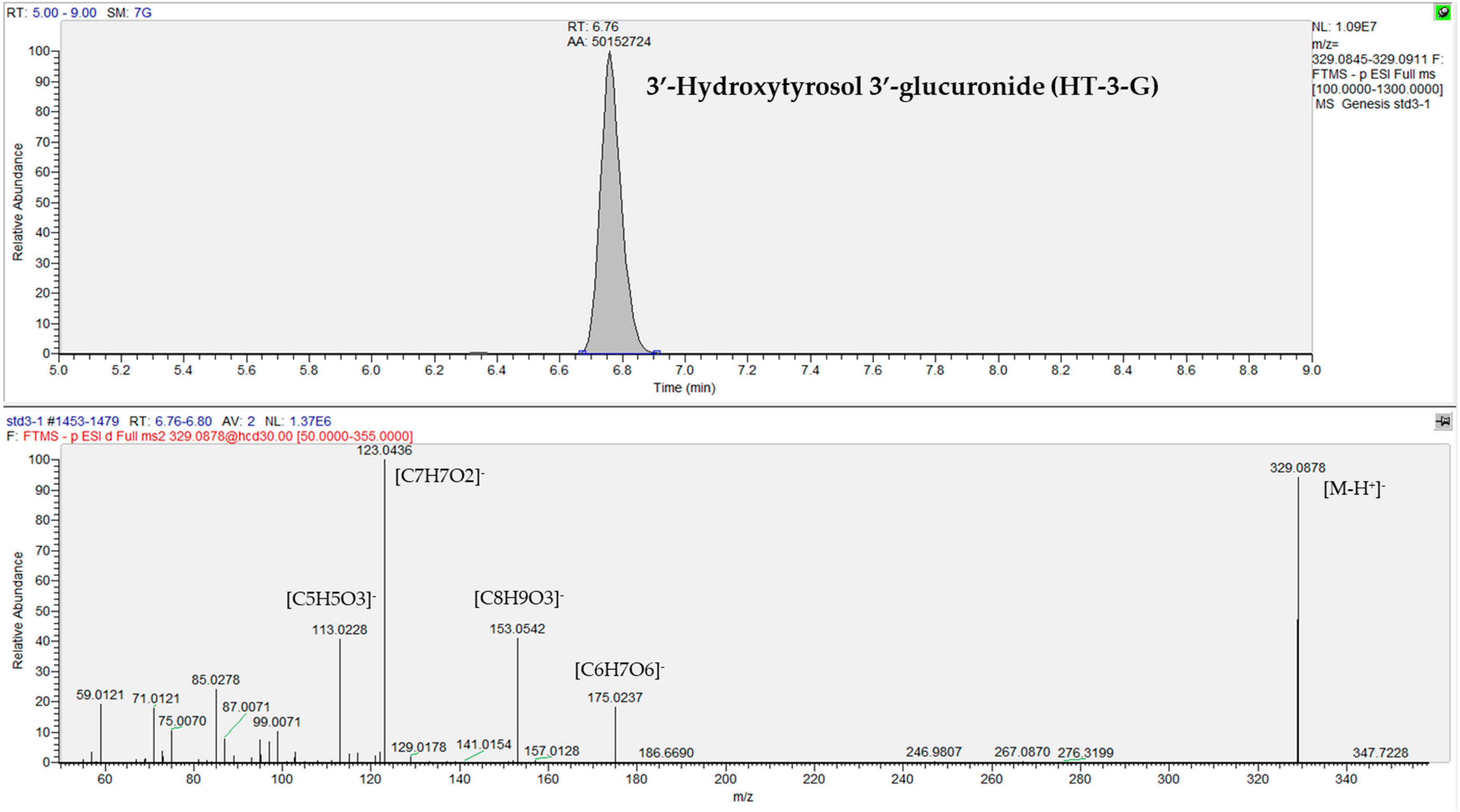

Figure S2 – LC-Q-Orbitrap chromatogram and MS² spectrum of HT-3-G

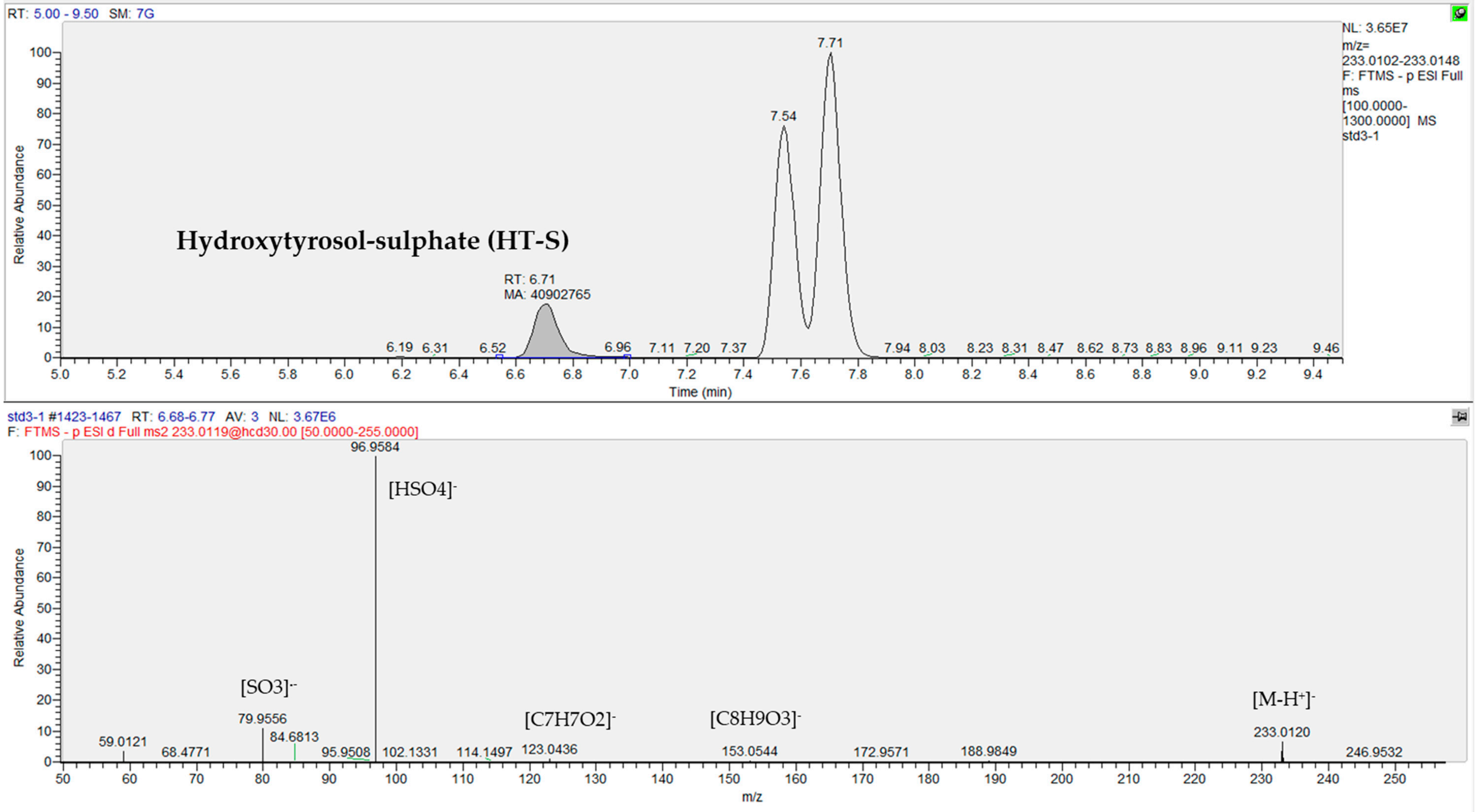

Figure S3 – LC-Q-Orbitrap chromatogram and MS² spectrum of HT-S

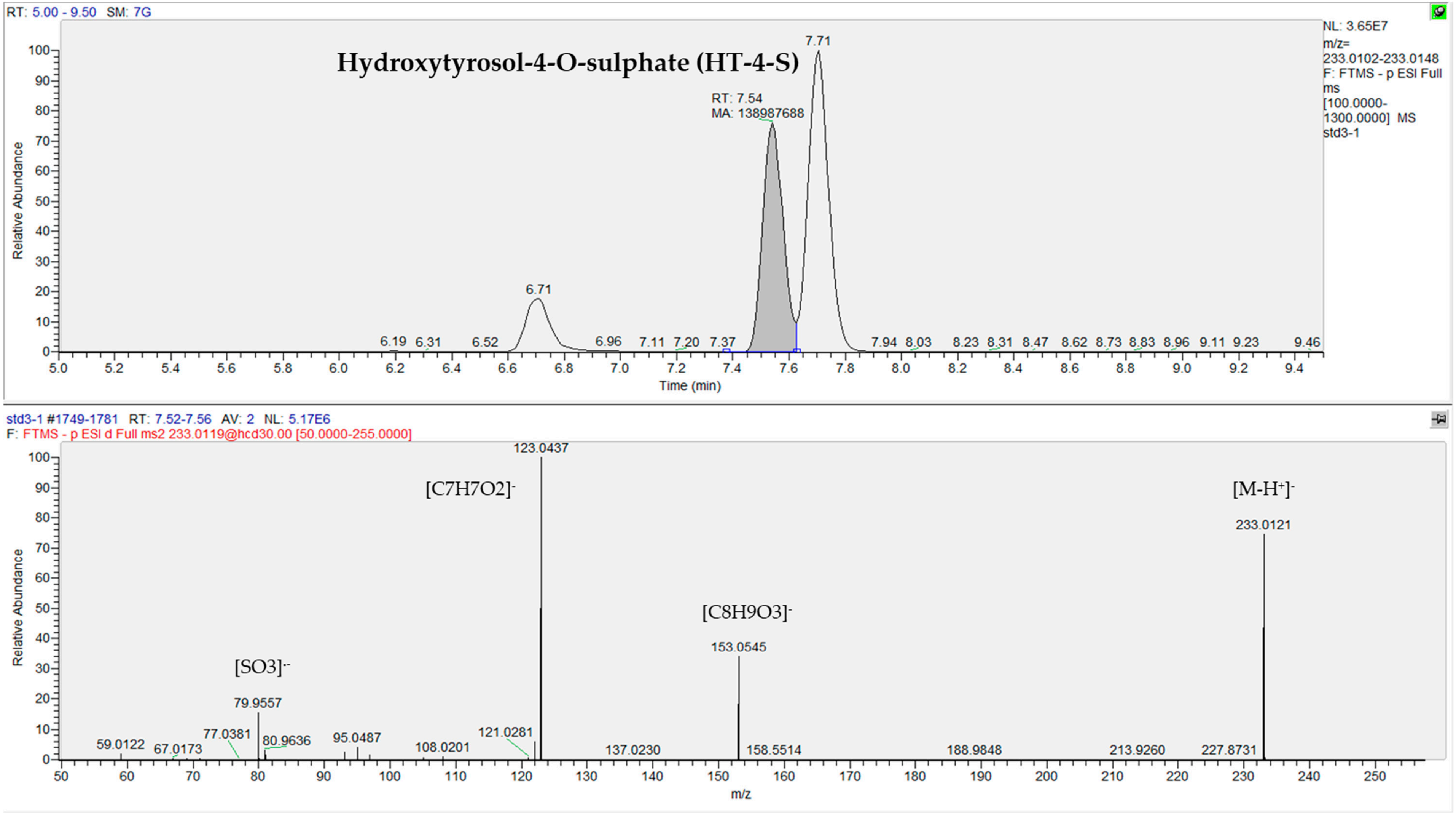

Figure S4 – LC-Q-Orbitrap chromatogram and MS² spectrum of HT-4-S

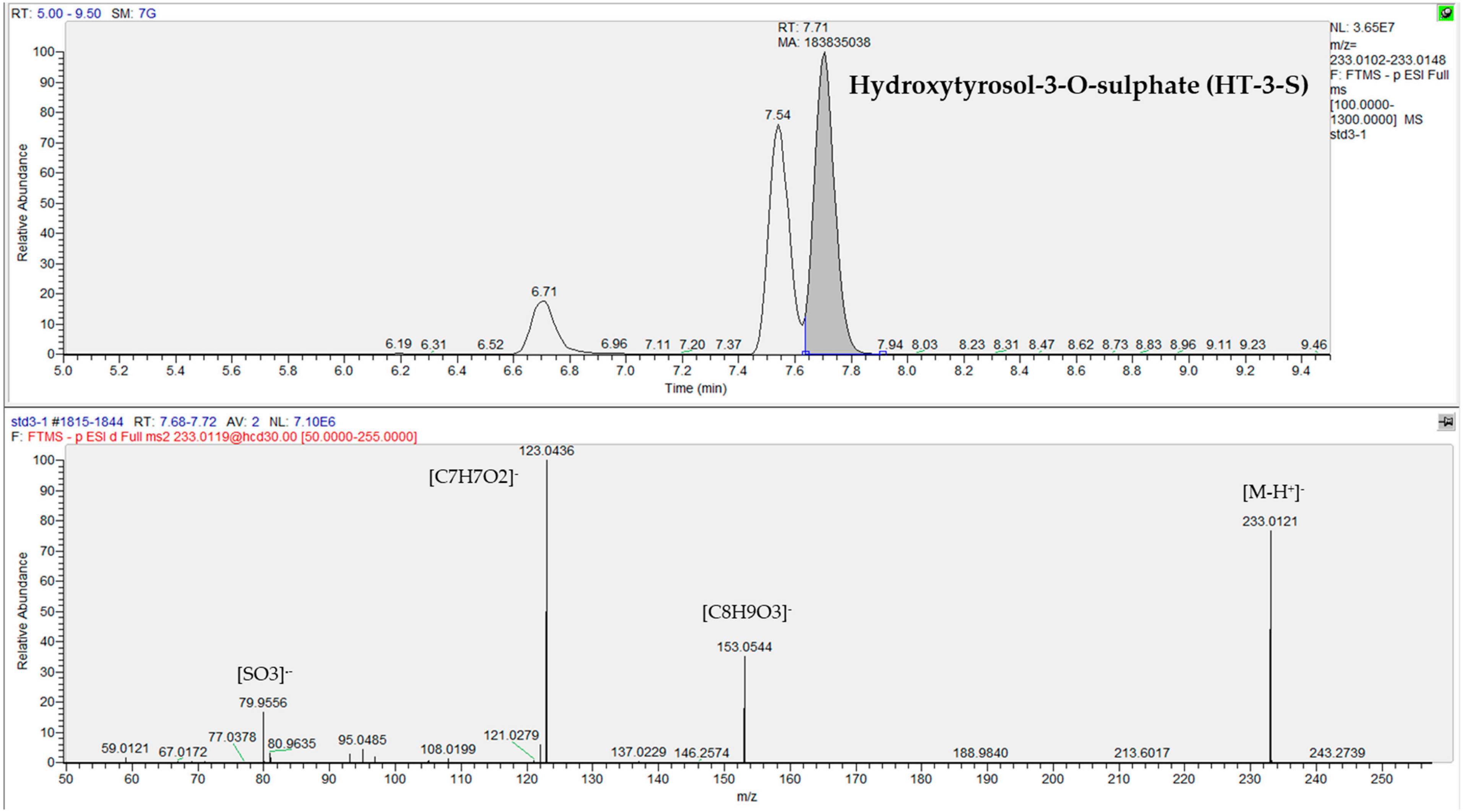

Figure S5 – LC-Q-Orbitrap chromatogram and MS² spectrum of HT-3-S

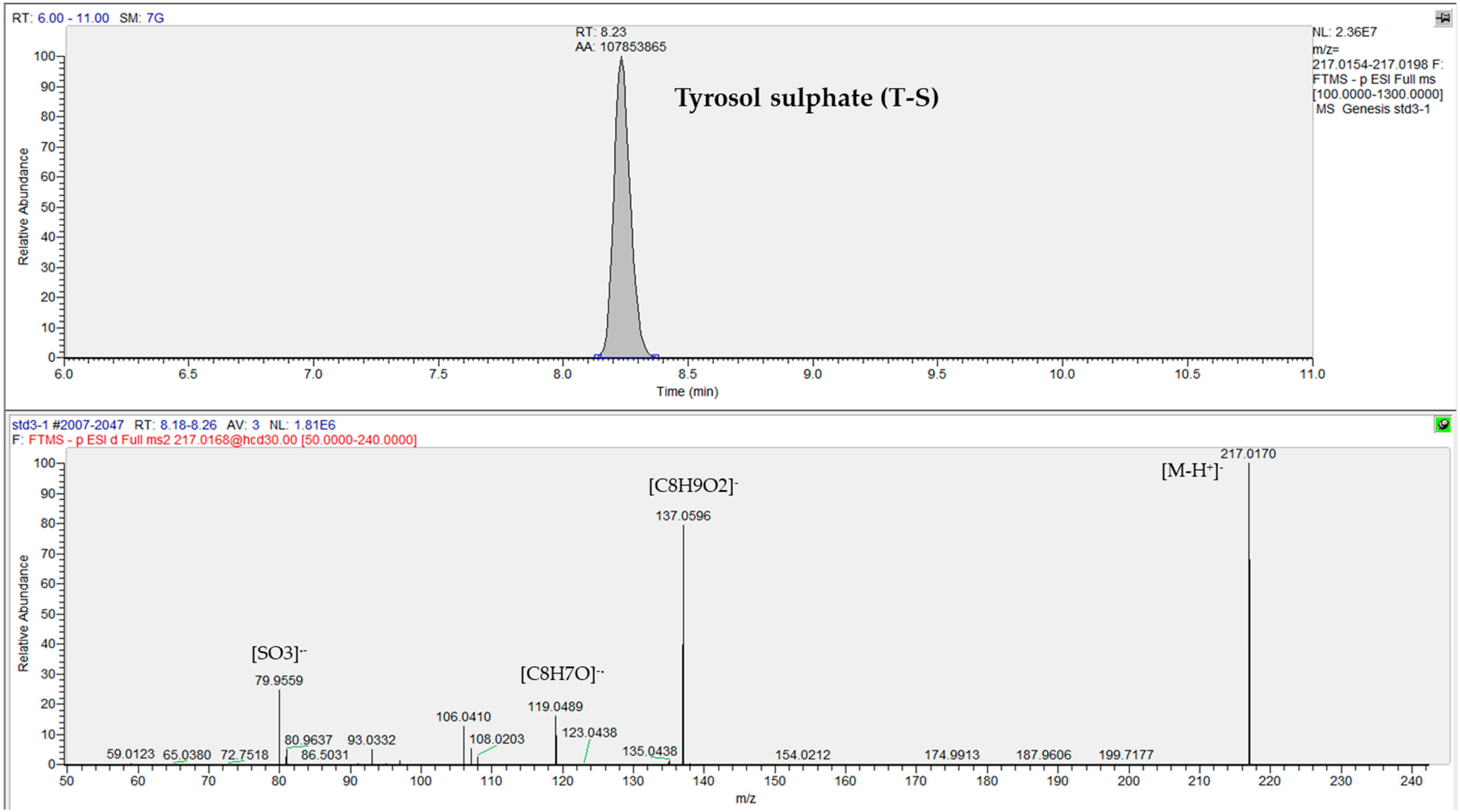

Figure S6 – LC-Q-Orbitrap chromatogram and MS² spectrum of T-S

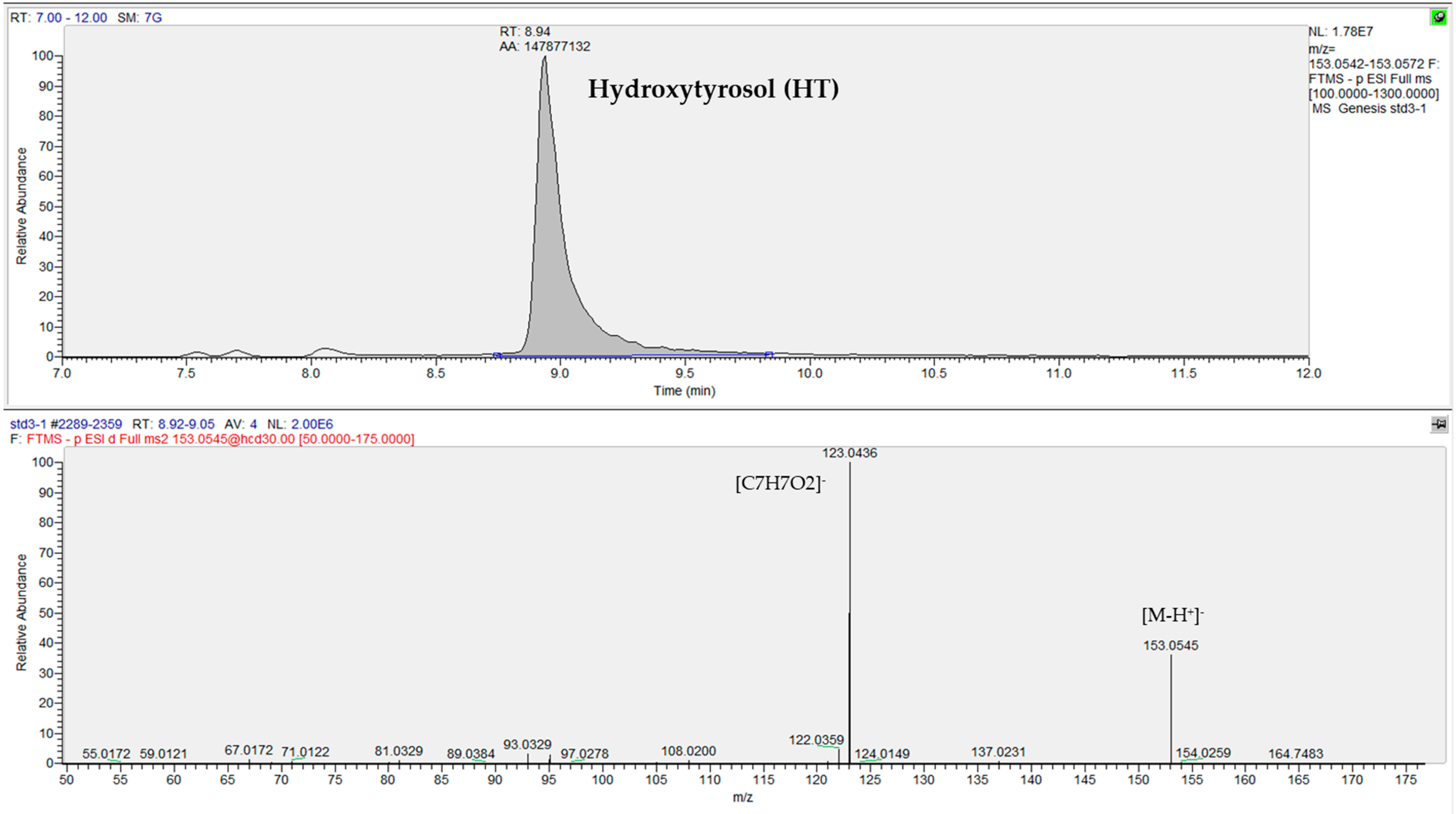

Figure S7 – LC-Q-Orbitrap chromatogram and MS² spectrum of HT

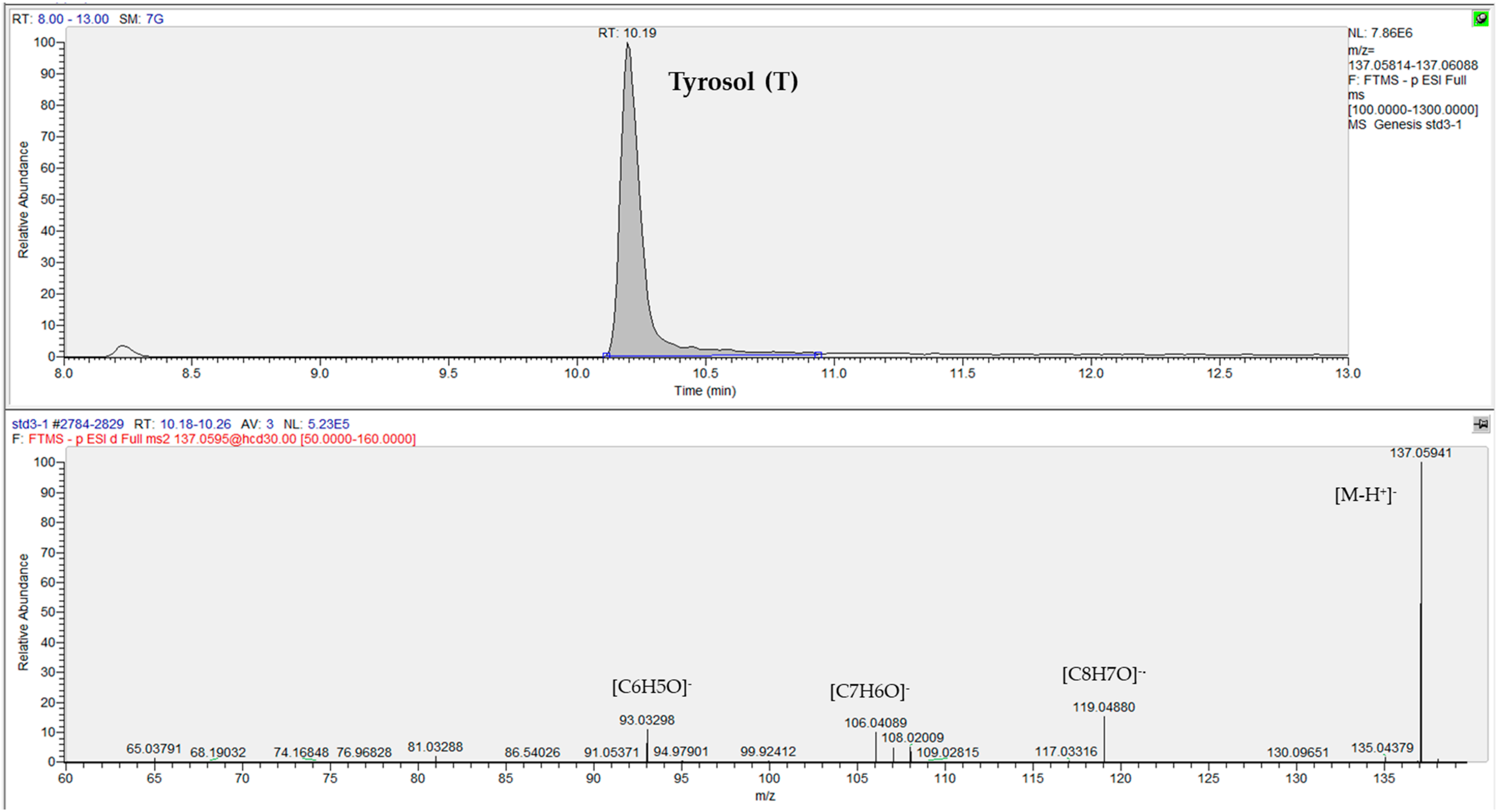

Figure S8 – LC-Q-Orbitrap chromatogram and MS² spectrum of T
